# Outbreaks of *Shigella sonnei* Infection with Decreased Susceptibility to Azithromycin Among Men Who Have Sex with Men — Chicago and Metropolitan Minneapolis-St. Paul, 2014

**Published:** 2015-06-05

**Authors:** Anna Bowen, Dana Eikmeier, Pamela Talley, Alicia Siston, Shamika Smith, Jacqueline Hurd, Kirk Smith, Fe Leano, Amelia Bicknese, J. Corbin Norton, Davina Campbell

**Affiliations:** 1 Division of Foodborne, Waterborne, and Environmental Diseases, National Center for Emerging, Zoonotic, and Infectious Diseases, CDC; 2 Minnesota Department of Health; 3 Epidemic Intelligence Service, CDC; 4 Chicago Department of Public Health, Illinois

Increasing rates of shigellosis among adult males, particularly men who have sex with men (MSM), have been documented in the United States, Canada, and Europe (1–4), and MSM appear to be at greater risk for infection with shigellae that are not susceptible to ciprofloxacin or azithromycin (5–8). Azithromycin is the first-line empiric antimicrobial treatment for shigellosis among children and is a second-line treatment among adults. Isolates collected in 2014 in two U.S. cities from outbreaks of shigellosis displayed highly similar pulsed-field gel electrophoresis (PFGE) patterns and decreased susceptibility to azithromycin (DSA). This report summarizes and compares the findings from investigations of the two outbreaks, which occurred among MSM in metropolitan Minneapolis-St. Paul, Minnesota, and Chicago, Illinois.

## Minneapolis-St. Paul

In February 2015, the Minnesota Department of Health Public Health Laboratory determined that 14 *Shigella sonnei* isolates obtained during May 13–December 8, 2014, displayed DSA (minimum inhibitory concentration >16 *μ*g/ml). CDC’s National Antimicrobial Resistance Monitoring System laboratory performed antimicrobial susceptibility testing and polymerase chain reaction testing to identify resistance genes on 13 of these isolates. All 13 isolates 1) were susceptible to nalidixic acid and ciprofloxacin, 2) were resistant to ampicillin and trimethoprim/sulfamethoxazole, 3) displayed DSA, and 4) harbored macrolide resistance genes *mph*A and *erm*B. The 14 isolates yielded five similar PFGE patterns (Figure).

Patients were male, had a median age of 39 years (range = 24–64 years) and lived in or near metropolitan Minneapolis-St. Paul. Patients were ill for a median of 12 days (range = 8–21 days), and one patient (7%) was hospitalized. Five were treated with ciprofloxacin, three with metronidazole, one with azithromycin, and one with an unknown antimicrobial agent. Of the four remaining patients, two were not treated with antimicrobial agents, and two had no available treatment information. Eight of nine with such information self-identified as MSM. Thirteen (93%) had received a diagnosis of sexually transmitted infection at least once during 2012–2015 (chlamydia [16 infections], gonorrhea [10], and syphilis [2]). Six (43%) were infected with human immunodeficiency virus (HIV); three had CD4 counts of 467, 516, and 899, respectively, in late 2014.

## Chicago

During July 31–October 31, 2014, the Chicago Department of Public Health detected 23 cases of *S. sonnei* infection among male Chicago residents aged >17 years. Among 17 (74%) isolates that underwent PFGE analysis, 10 displayed patterns highly similar to or indistinguishable from patterns in the Minneapolis-St. Paul outbreak (Figure) and are included in this analysis. The CDC laboratory performed antimicrobial susceptibility testing on eight Chicago isolates; all eight displayed the same antimicrobial susceptibility profile as the Minneapolis-St. Paul isolates and harbored *mph*A and *erm*B. The median age of the Chicago patients was 35 years (range = 24–53 years). Seven (88%) patients self-identified as MSM among eight who provided this information, and six (60%) were infected with HIV. Five (50%) patients were hospitalized; HIV infection was not associated with hospitalization (Fisher’s exact test, p = 0.5).

## San Francisco and Los Angeles

Using CDC’s PulseNet, investigators detected additional isolates with PFGE patterns indistinguishable from the outbreak clusters. A man aged 32 years from San Francisco who self-identified as MSM and reported no travel developed illness in January 2015 (Figure). In addition, one PFGE pattern was associated with a previously reported 2012 outbreak of 43 cases of shigellosis with DSA in Los Angeles (Figure) (9).

## Further Laboratory Findings

To better understand the prevalence of DSA among *Shigella* in Minnesota, the public health laboratory tested the 80 (86%) available isolates from the 93 shigellosis cases reported in Minnesota in 2014. In addition to the 14 outbreak-associated *S. sonnei* isolates with DSA, the public health laboratory found DSA in two nonoutbreak *S. sonnei* isolates and four *Shigella flexneri* isolates, for a total of 20 (25%) shigellae with DSA in Minnesota in 2014. Patients infected with *Shigella* with DSA had a median age of 38 years (range = 19–64 years), 18 (90%) were male, and nine (45%) were known to be infected with HIV. Among 16 male patients who provided travel information, none reported recent international travel; two female patients reported travel to Asia.

MSM in the United States and abroad appear to be at greater risk for shigellosis with DSA (10). MSM can protect themselves and others from shigellosis by washing hands before preparing food or eating and after using the toilet; refraining from swimming for 1 week after recovering from shigellosis; avoiding sex while they or their partners have diarrhea and for a few weeks after recovering from shigellosis; washing hands, genitals, and anus before and after sex; and using barriers such as dental dams and gloves during anal rimming and fisting. Clinicians should obtain stool cultures from patients with symptoms of shigellosis and choose treatments, when needed, based on isolate antimicrobial susceptibility profiles. Clinical guidance for the testing and interpretation of azithromycin susceptibility among shigellae is needed to guide patient management. Increasing rates of routine or outbreak-driven PFGE testing of shigellae can help track *Shigella* strains with DSA.

## Figures and Tables

**FIGURE:**
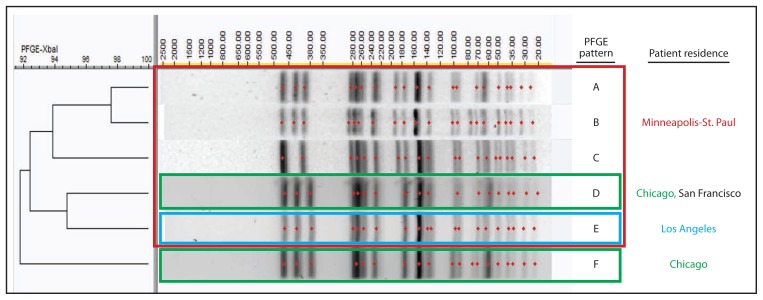
Pulsed-field gel electrophoresis (PFGE) patterns created using enzyme *Xba*I and associated with outbreaks during 2014–2015 of *Shigella sonnei* infection with decreased susceptibility to azithromycin among men who have sex with men in 1) metropolitan Minneapolis-St. Paul, Minnesota (patterns A–E); 2) Chicago, Illinois (D and F); and 3) San Francisco, California (D); as well as with a 2012 outbreak in 4) Los Angeles, California (E)
